# Analysis of a Community Pharmacy Intervention to Improve Low Adherence Rates to Oral Diabetes Medications

**DOI:** 10.3390/pharmacy5040058

**Published:** 2017-10-19

**Authors:** Jerica Singleton, Stevie Veach, Christine Catney, Matthew Witry

**Affiliations:** 1 Department of Pharmacy Practice and Science, University of Iowa College of Pharmacy, PGY1 Community Pharmacy Practice at Time of Study, Iowa City, IA 52242, USA; 2 Department of Pharmacy Practice and Science, University of Iowa College of Pharmacy, Iowa City, IA 52242, USA; stevie-veach@uiowa.edu (S.V.); christine-catney@uiowa.edu (C.C.)

**Keywords:** diabetes, medication adherence, proportion of days covered

## Abstract

For patients with diabetes, suboptimal medication adherence contributes to disease progression, complications, and increased healthcare costs. Identification of, and intervention for patient-identified reasons for nonadherence are essential to improving medication adherence. This prospective, quality improvement study was conducted at an independent community pharmacy in the Mid-West United States. Patients with a proportion of days covered (PDC) for their oral antidiabetic medications of less than 80% were contacted by telephone and interviewed by a clinical pharmacist. The interviews and corresponding adherence interventions were guided by an abbreviated version of the Drug Adherence Work-Up (DRAW©) tool that focused on oral medications for diabetes. The change in PDC 120-days post-interview was assessed to determine the change in adherence rates. Patients receiving the pharmacist-delivered adherence intervention had significantly higher 120 day PDC values which are likely to indicate more regular medication-taking at home. Almost half of study patients signed up for medication synchronization and these patients trended toward higher PDC values, although the relative difference was not statistically significant from those receiving the intervention and not opting to have their medications synchronized.

## 1. Introduction

As of 2015, over 30.3 million Americans have a diabetes diagnosis, and another 84.1 million have prediabetes. In 2012, total direct and indirect costs of healthcare for patients with diabetes was estimated at $245 billion with average annual medical expenses of $13,700 per patient. In 2014 alone, 14.2 million emergency room visits and 7.2 million hospitalizations were for patients with diabetes [1]. While diabetes prevention is important, for those already afflicted, adherence to recommended medications can lower the risk of being hospitalized or visiting the emergency room by 13% [2].

Medication non-adherence is common among patients with type 2 diabetes who are taking oral antidiabetic medications. Studies have reported rates that range anywhere from 36% to 93% medication adherence for oral antidiabetic medications [3]. Common reasons for medication non-adherence among patients with diabetes include forgetfulness, concerns about side effects or harm from medicines, and cost [4,5]. The stigma associated with taking medications, paired with the fear of medication-related complications can cause apprehension about taking medications as prescribed [4,5]. Medication non-adherence also is associated with higher direct and indirect healthcare costs [6].

There are a variety of interventions that have been used to improve adherence [7]. Successful interventions generally involve a multifaceted approach that uses a combination of behavioral interventions, disease-state education, medication organization aids, and technological supports [8]. Adherence scales have been used to identify specific reasons for non-adherence [9]. However, a limitation of these scales is many do not provide guidance to the provider about strategies to resolve the identified barriers to adherence. The Drug Adherence Work-Up (DRAW©) tool provides this type of guidance in an interview guide format with recommended targeted action for the provider to consider [10].

The objective of this this study was to pilot a telephone intervention for providing targeted adherence interventions to patients taking oral medicines for diabetes. The justification for focusing on diabetes is that non-adherence is common for this condition and medication adherence is important to avoiding or delaying diabetes disease progression.

## 2. Materials and Methods

### 2.1. Study Design

This study was a prospective, pharmacist-initiated, quality improvement initiative that used a pre-post comparison where patients served as their own comparator. The study was conducted at a single site of a small independent pharmacy chain in the Mid-West, United States and occurred over a year-long PGY1 Community Pharmacy Residency. Interventions occurred from 30 September 2016, to 7 February 2017. The University of Iowa Institutional Review Board approved the project as a quality-improvement study.

### 2.2. Patients

At the initiation of the study, a query using a third party software platform for facilitating pharmacy quality improvement identified 96 patients taking at least one oral antidiabetic medication and who also had a proportion of days covered (PDC) for their oral antidiabetic medications of less than 80%. Proportion of days covered is a standard measure for medication adherence that makes important adjustments to avoid over-estimation [11]. The threshold of 80% is generally accepted as appropriate for chronic conditions such as diabetes [11]. Patients were excluded if they used more than one pharmacy for their prescriptions, stopped taking oral medications for diabetes during the study period, or if they were not able to communicate via telephone in English. All patients interviewed were patients that regularly obtained their prescriptions from the study pharmacy and this was verified using dispensing records. If, when the pharmacist called to deliver the intervention the patient was no longer taking the medication, she was thanked for her time and told the pharmacy would update their records. Demographic information including age, gender, insurance type, number and type of oral antidiabetic medications was collected for patients who completed the study using information from the dispensing system for the purpose of describing the sample.

### 2.3. Procedure

Eligible patients were contacted via their listed pharmacy profile telephone number up to 3 times. Interviews were performed by the pharmacy resident and guided by an abbreviated version of the DRAW© tool (Appendix A) specific for oral antidiabetic medications. The rationale for creating an abbreviated version of the DRAW tool was to streamline the interview process because the original version asked about the adherence domains in multiple ways. The abbreviated version only asked about each domain once. This reduced the redundancy of the questions and the duration of the calls while still addressing the major types of adherence barriers (forgetfulness, side effects, need perception, and cost). The tool retained its list of targeted interventions based on the particular adherence barrier (e.g., medication box for forgetfulness, motivational interviewing techniques for low perceived medication need. This work-up was tailored towards oral antidiabetic medications taken by the patient with diabetes. The pharmacist making the telephone intervention used paper forms to record the problems and interventions identified during the call. These were later transcribed into an Excel Spreadsheet (Redmond, WA, USA). Phone interviews lasted from 5 to 10 min per patients.

### 2.4. Outcomes

The primary outcome of this study was the change in PDC for oral antidiabetic medications following the telephone intervention. Patients served as their own control. Pre-interview PDCs were collected from the third-party software connected to the dispensing system for the pharmacy on 30 September 2016. These baseline PDCs were calculated based on refill history for the past 270 days, or when the medication was initiated, if sooner. Post-interview PDCs were assessed 120 days following the interaction between pharmacist and patient using the third-party software. In some instances, manual PDCs had to be calculated such as when patients had cash transactions that did not go through insurance. For manual PDC calculations, patient profiles were analyzed looking at fill dates for all oral antidiabetic medications for the needed period. The number of days in the period was adjusted if patients began an oral antidiabetic regimen after the standard period length. For patients taking multiple oral antidiabetic medications, the PDC was calculated based on if at least one medication provided coverage during the designated period as defined by the Pharmacy Quality Alliance [11]. For each patient, a timeline was drawn to the lay out when the medication fills occurred, allowing for the period and covered days to be calculated. If a patient had an oral antidiabetic medication on hand every day of the period, then their number of days covered equaled their number of days in the period resulting in a 100% PDC. The following equation was used for manual PDC calculations. This involved truncating fills or moving covered days forward if necessary to provide a valid estimate of covered days [8].

$$\mathrm{PDC}=\left(\frac{\mathrm{Number}\;\mathrm{of}\;\mathrm{days}\;\mathrm{in}\;\mathrm{period}\;\mathrm{covered}}{\mathrm{Number}\;\mathrm{of}\;\mathrm{days}\;\mathrm{in}\;\mathrm{period}}\right)\times 100$$

The secondary outcome was to characterize the reasons for non-adherence and pharmacist interventions made during the telephone interviews. The reasons and interventions were abstracted from the paper forms completed by the pharmacist and assigned to the categories contained in the DRAW tool [10]. The corresponding text accompanied the categories.

### 2.5. Analysis

Data were entered and managed using Microsoft Excel (Redmond, WA, USA). Demographics for study variables were calculated. A paired t-test (2-sided) was used to test for a difference in PDCs from the 270-day before interview period and the 120-day post interview period using an alpha of 0.05. Statistical analyses were performed in SPSS v 24 (Chicago, IL, USA). Frequencies were calculated for the various categories of reasons for non-adherence and interventions contained in the completed DRAW tools. Text in addition to the DRAW categories was examined to identify additional detail about the identified reasons for non-adherence and corresponding interventions for the purpose of adding context to the quality improvement effort.

## 3. Results

**Figure 1:**
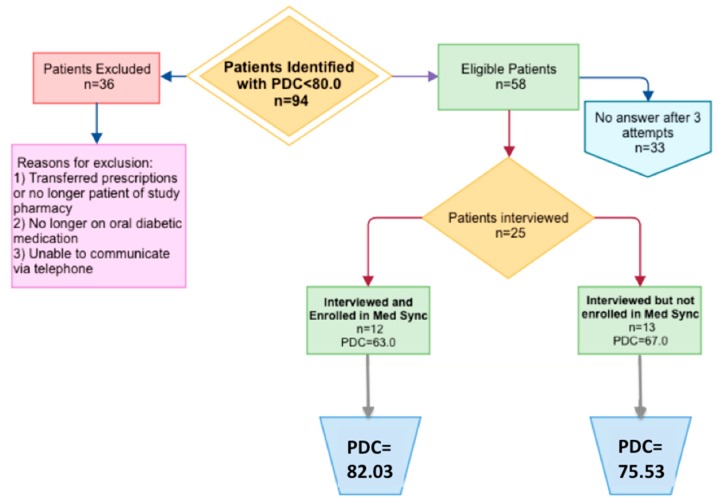
Graphical Representation of Patient Flow.

An investigation of patient medication profiles and refill histories resulted in the exclusion of 36 patients from the initial pool of 96patients. This included patients who were no longer taking oral antidiabetic medications, used a different pharmacy, or had documented barriers to participating in a telephone interview. The remaining 58 patients were called via telephone, 33 did not answer after three attempts and 25 and were interviewed (Figure 1). Demographic information of the patients interviewed is found in Table 1.

**Figure 2:**
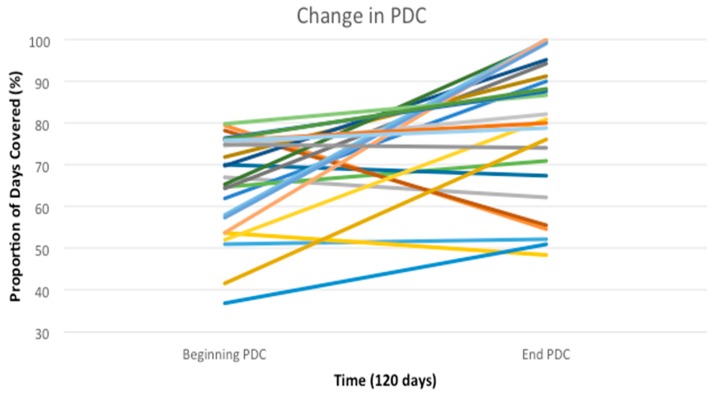
Change in PDC over 120 days. Each line represents one patient in the study.

The average baseline PDC (*n* = 25) was 65.2%. The average 120-day post interview PDC rose to 78.7%, a 13.5% increase (*p* < 0.002), see Table 2. The cohort also had 12 patients reach the threshold PDC value of 80% which is considered to be adherent. Two patients achieved a PDC value of 100%, up from 58% and 54%, respectively, see Figure 2.

A simple majority, 52% (*n* = 13) of patients self-identified at least one reason for nonadherence in response to the prompts from the DRAW© tool interview, and 12 reported no barriers. Of those reporting a reason, most (69%) stated forgetfulness. Other recognized reasons for non-adherence include: feeling as though the medication is not helping with their disease state, experiencing side effects from their medication(s), and refill complications either at the pharmacy or from their provider, see Table 3.

Following the identification of reasons for non-adherence, the pharmacist offered targeted interventions at their discretion to 18 patients (72%) with some receiving more than one intervention. There was an average of 1.6 interventions per patients performed. Recommended interventions were detailed on the abbreviated DRAW^®^ tool followed during interview. Of the 12 patients who did not identify a reason for non-adherence, 6 patients (50%) agreed to an intervention during the interview; including medication box counseling to all 6 patients with four of these patients enrolling in the pharmacy’s medication synchronization program.

Patients were eligible for medication synchronization if they filled more than three prescriptions on more than two separate days in a 30-day period as determined by third-party software. Seventeen patients were eligible and offered medication synchronization during interview. Of those receiving any intervention, two-thirds (*n* = 12) were enrolled in medication synchronization. Other interventions are detailed in Table 4.

Patients undergoing medication synchronization experienced an average increase change in PDC of 18.8%; resulting from a starting average PDC of 63.2% and an ending PDC of 82%. Patients who were not eligible for medication synchronization or who chose not to participate experience an average increase change in PDC of 8.5%; resulting from a starting average PDC of 67.1% and an ending PDC of 75.5%, see Table 5. This difference, however, was not statistically significant.

## 4. Discussion

The study supports the role of community pharmacists in improving the medication adherence of patients with diabetes. This particular interview approach was associated with an average increase in PDC of 13.5%, 72% of patients having an increase in PDC, and 48% achieving a PDC of >80–threshold associated with acceptable adherence [11].

The most common intervention in this telephone based interview program was to initiate medication synchronization. This process aligns a patient’s chronic medication refills so they all can be picked up at the same time. This option is beneficial for some patients, but may not work for everyone’s needs, as evident by a small number of patients not being interested in the service when offered. The analysis suggested that medication synchronization may be associated with a greater increase in PDC over a 120-day post-intervention period compared to patients who did not participate in medication synchronization, although statistical significance of the difference was not established for this small sample. Such a comparison may be problematic due to selection bias that can occur with voluntary enrollment into this service. Patients that sign up to have their medications synchronized may have different medication beliefs, practices, and other characteristics than those that decline the service. For example, the medication synchronization patients in our study started with a lower average PDCs, so they had more room to improve.

About half of patients participating in the pharmacist interview did not identify a reason for non-adherence despite their being targeted by the software as having evidence of non-adherence. This inconsistency may be due to social desirability bias or the patient not wanting to discuss adherence with a pharmacist or over the telephone. Alternatively, the patient may have had an adherence barrier that was not prompted by the interview guide. Alternatively, a logistical reason related to the days supply of medication, the doctor having given new instructions without a new prescription, or some other situation may have resulted in an inaccurate adherence alert [12]. Regardless, several patients for whom a barrier was not identified agreed to an organization-related intervention such as a medication box or enrolling in the pharmacy’s medication synchronization program.

This pilot and feasibility study had several limitations. First, this study lacked a control group and therefore it cannot be determined if the adherence changes were solely due to the intervention or other influences. Also, a study of 1 pharmacy location with a small sample limits generalizability. More research in different settings with different patients is needed. Future research may include comparing the abbreviated DRAW© with the original DRAW© tool or other iterations like the M-DRAW [13]. Comparisons also are needed with control groups and comparing in-person to telephone interviews. It also may be beneficial to co-administer the DRAW© in combination with a service like diabetes education or other appointment based interventions like comprehensive medication reviews. Also, the questions from the DRAW© tool could be incorporated in routine counseling as pharmacists become better versed on the variety of reasons why patients may be non-adherent. Regardless of the approach, discussing adherence and medication use is an important role for pharmacists, warranting expansion and further optimization in the community pharmacy setting.

## 5. Conclusions

This pharmacy-based, telephonic adherence intervention focused on patients with diabetes who were non-adherent to their diabetes medications. The clinical pharmacist used a newly developed abbreviated version of the DRAW© tool to identify patient reasons for non-adherence and recommend interventions such as medication synchronization and targeted counseling. Patients receiving the intervention had a significant improvement in adherence over the study period as measured by PDCs.

## Figures and Tables

**Table 1:** Demographic and clinical characteristics of patients (*n* = 25).

**Age (years)**	**Number (%)**
20–29	2 (8%)
30–39	3 (12%)
40–49	5 (20%)
50–59	11 (44%)
60–69	3 (12%)
70–79	1 (4%)
**Gender**	**Number (%)**
Male	14 (56%)
Female	11 (44%)
**Insurance**	**Number (%)**
Medicaid only	11 (44%)
Medicare part D only	1 (4%)
Private Insurance and Medicaid secondary	1 (4%)
Private Insurance only	9 (36%)
Cash Paying Patient	3 (12%)
**Number of Oral Antidiabetic Medications**	**Number (%)**
1	21 (84%)
2	0 (0%)
3	3 (12%)
**Type of Oral Antidiabetic Medications**	**Number (%)**
Biguanide	19 (76%)
Sulfonylurea	4 (16%)
Thiazolidinedione	2 (8%)

**Table 2:** Change in PDC results (*n* = 25).

PDC	Beginning of Study	End of Study
30–40%	1	0
40–50%	1	1
50–60%	6	4
60–70%	7	2
70–80%	10	5
80–90%	0	5
90–100%	0	8

**Table 3:** Patient identified reasons for medication nonadherence ^1^.

Identified Reason for Non-adherence	Number of Patients
Sometimes forgets	9
Refill complications	2
Feels as though medications are not helping	1
Stopped due to side effects	1
No barrier identified	12

^1^ Each adherence workup could be associated with more than one reason for nonadherence.

**Table 4:** Interventions performed during patient interviews.

DRAW Intervention Category	Intervention Performed	Number of Patients ^1^
Reminder Tools	Medication Synchronization	12
	Medication Box	7
Reminder Tools	Helped set up Smart Phone Reminders	1
Patient Education	Discussed role and importance of medication in diabetes control	3
Patient Education	Tips for avoiding side effects	1
Patient Education	Exercise and diet counseling	4
Cost Reduction	Alternative Medications ^2^	1
None	No adherence actions needed	7

^1^ Each adherence workup could be associated with more than one intervention. ^2^ Changing medication from Immediate Release to Extended Release was suggested to the patient, and prescriber consultation was offered due to side effects experienced.

**Table 5:** Comparison of change in PDC for adherence intervention.

	PDC	Beginning of Study	End of Study
Patients undergoing medication synchronization (*n* = 12)	<30%	-	-
	30–40%	-	-
	41–50%	1	1
	51–60%	3	-
	61–70%	5	1
	71–80%	3	3
	81–90%	-	2
	91–100%	-	5
Patients not participation in medication synchronization (*n* = 13)	<30%	-	-
	30–40%	1	-
	41–50%	-	-
	51–60%	3	4
	61–70%	2	1
	71–80%	7	2
	81–90%	-	3
	91–100%	-	3
